# Expression and purification of recombinant HTLV-I/-II linear epitopes antigen and its application for screening of suspected patients

**Published:** 2017-02

**Authors:** Roxana Faramarzi, Samaneh Dolatabadi

**Affiliations:** Department of Microbiology, Neyshabur Branch, Islamic Azad University, Neyshabur, Khorasan Razavi, Iran

**Keywords:** HTLV, Chimeric antigen, Protein expression, Purification

## Abstract

**Background and Objectives:**

The linear epitopes of gp46-I, gp46-II, gp21 and p19 are used in diagnosis of HTLV-I/-II infections. The aims of this study was to obtain high-level expression and purification of recombinant antigen (RA) containing these epitopes. Large-scale preparation of such antigen probably worths for diagnostic purpose.

**Materials and Methods:**

The synthetic DNA encoding RA was synthesized and over-expressed as soluble in *Escherichia coli* BL21 (DE3) cells. Expression and distribution of the His-GST-RA protein were evaluated using SDS-PAGE. The soluble RA was purified utilizing Ni-NTA agarose beads under native conditions and was concentrated by ultra filtration. Using 20 sera specimens from HTLV infected patients, the antigenicity of the purified protein was confirmed in ELISA and western blotting analysis.

**Results:**

SDS-PAGE revealed that the purified protein was more than 90% pure. The final yield was approximately 25 mg per liter of culture medium. ELISA results showed that RA could specifically bind to anti-HTLV-I/-II antibodies in infected sera.

**Conclusion:**

RA could be a candidate for HTLV-I/-II screening and the strategy presented in this study could be used for easy production of this diagnostic protein.

## INTRODUCTION

Human T-lymphotropic virus type I (HTLV-I) is a retrovirus known to be the etiological agent of adult T-cell leukemia/lymphoma (ATLL) and myelopathy/tropical spastic paraparesis (HAM/TSP) diseases (1). Human T-lymphotropic virus type II (HTLV-II) is closely related to HTLV-I which shares approximately 70% genome homology with HTLV-I (2). In contrast to HTLV-I, the role of HTLV-II in development of neurological diseases is less clear (2, 3).

HTLV-I and -II infection is asymptomatic in more than 90% of cases, most of them are completely unaware of their infection. Screening of asymptomatic carriers is the main strategy to prevent infections with HTLV-I and -II and spreading (4). The most cost-effective screening method for detection of HTLV-I and -II antibodies from sera samples is enzyme-linked immunosorbent assay (ELISA) (5).

Whole viral lysate, full length antigen and synthetic peptides are used as capture antigen for detection of HTLV antibodies. Viral lysate and full length antigens give some non-specific interaction with antibodies arises against other viral infections (5). Therefore there is a tendency for use of short length peptides antigen (6, 7). At least three peptide antigens should be used for accurate and precise detection of HTLV-I and HTLV-II antibodies (5). This can decrease the efficiency of detection by non-equivalent binding of peptides antigens, competition for binding to solid surface and waste of peptides during washing steps. Use of single chimeric antigen containing major epitopes could resolve these problems and improve the sensitivity and specificity of the tests (7, 8).

In previous study, Heydari-Zarnagh designed a recombinant protein (named SCA) for specific detection of HTLV-I antibodies incorporating linear epitopes of gp46, gp21 and p19 and flexible linker as spacer arm between epitopes (9). In this study we aimed to add specific epitope of HTLV-II to SCA and construct a recombinant antigen for simultaneous detection of HTLV-I and HTLV-II antibodies.

## MATERIALS AND METHODS

All reagents used in the present study were purchased from Sigma (Missouri, USA) unless otherwise indicated. The oligonucleotides were synthesized by Biomatik Company (Wilmington, USA). Restriction enzymes were purchased from Takara (Kyoto, Japan); plasmid DNA extraction and gel purification kits were obtained from iNtRON (Daejoen, Korea); T4 ligase, DNA marker, and IPTG were purchased from Fermentas (Opelstr, Germany); protein marker was purchased from Cinaclone (Tehran, Iran); and Ni-NTA agarose resin was obtained from Qiagen (Valencia, USA).

### Serum samples.

Serum samples were collected from 10 patients with HTLV-I (anti-HTLV-I positive) and 10 patients with HTLV-II (anti-HTLV-II positive) infection referred to Razavi hospital, Mashhad city, Iran. All samples were diagnosed as positive when tested with commercial ELISA kits (MP Diagnostics™; HTLV-I/II ELISA 4.0). Samples from 20 healthy volunteers were used as controls and tested with ELISA.

### Recombinant antigen (RA) design.

The amino acid sequence of immunodominant peptides derived from HTLV-I/-II diagnostic proteins [162-214 of gp46-I from HTLV-I (LLVDAPGYDPIWFLNTEPSQLPPTAPPLLPHSNLDHILEPSIPWKSKL), 160–210 of gp46-II from HTLV-II (DAPGYDPLWFITSEPTQPPPTSPPLVHDSDLEHVLTPSTSWTTK), 100–130 of p19 (PPPPSSPTHDPPDSDPQIPPPYVEPTAPQVL) and 370–400 of gp21 (KIAQYAAQNRRGLDLLFWEQGGLCKALQEQCCFLNITNSHV)] from both HTLV-I/II were connected together using flexible linker (amino acid sequence: EAAAK). The selected epitopes were investigated further using multiple sequence alignments tools. All sequence-profile alignments were performed using a standard pairwise alignment of Clustal Omega. The resulting chimeric protein was reversely translated (http://www.bioinformatics.org/sms2/rev_trans.html) and optimized according to *E. coli* codon usage by JAVA codon optimization tool (http://www.jcat.de/) and analyzed by rare codon analysis tool (https://www.genscript.com/tools/rare-codon-analysis). The *EcoRI* and *BamHI* restriction sites were inserted into the ends of DNA sequence for cloning purpose.

### Construction of the expression plasmid.

The synthetic gene was sub-cloned into the *BamHI/Eco-RI* sites of a pGS21a vector to produce pGS21a-RA according to standard DNA manipulation methods. Accuracy of sub-cloning was verified using DNA sequencing. Then, the recombinant plasmid was transformed into chemically competent *E. coli* BL21 (DE3) cells and the colonies were selected on a LB plate supplemented with 100 μg/ml ampicillin.

### Expression of the recombinant antigen.

An *E. coli* BL21 (DE3) colony harboring pGS21a-RA expression vector was grown overnight at 37°C in 5 ml of LB medium with 100μg/ml ampicillin. This culture was inoculated into a baffled flask that contained 100ml of the LB medium with 100μg/ml ampicillin. The culture was incubated at 37°C until an OD_600_ of 0.6 was reached. Expression was induced by adding IPTG (final concentration of 0.4mM) and incubating the cells at 18°C for an additional 12h under constant shaking at 250 rpm. The cells were harvested by centrifugation at 5000 rpm for 10min at 4°C. The pellets were resuspended in PBS and mixed with 2× sample loading buffer heated at 100°C for 15 min. The samples were analyzed by SDS-PAGE in 12% polyacrylamide gels. The non-induced controls (as negative controls) were analyzed in parallel.

### Solubility determination.

To check the solubility of His-GST-RA under conditions for expression, the *E. coli* pellets obtained from 50 ml of culture following IPTG induction were re-suspended in phosphate buffered saline (PBS) buffer (137 mM NaCl; 2.7 mM KCl; 10 mM Na_2_HPO_4_.2H_2_O; 2 mM KH_2_PO_4_, pH 7.4) and subjected to sonication (Bandelin, Germany) on ice. The sonication was done at a frequency of 20 kHz for 2 minutes, bacteria were pulsed for 6 × 10 s intervals. After sonication, the cell lysate was centrifuged at 12,000 rpm for 20 min at 4°C. The clear supernatant and pellets were analyzed by SDS-PAGE in 12% polyacrylamide gels.

### Simple purification of RA.

The supernatant was centrifuged at 12,000 rpm for 15 min at 4°C and filtered through a 0.22-μM filter to remove any insoluble material. Subsequently, the cell lysate supernatant was transferred into a 1 × 5-cm column packed with 1 ml of Ni^+^-NTA resin and protein was purified according to the manufacture instructions. A flow rate of 0.5 ml/min was used in all the chromatographic steps. The protein concentration was determined using the Bradford method (10). The purity of the concentrated RA was analyzed by 12% SDS-PAGE.

### Western blot analysis of the recombinant RA.

The purified RA was separated on 12% SDS-PAGE under reducing conditions and then blotted to nitro-cellulose membrane. The membrane was blocked with blocking buffer (PBS, 0.5% Tween-20, and 5% non-fat dry milk powder) and then incubated overnight with serum containing HTLV antibodies at 4°C. After washing, the immunoreactive bands were visualized using horseradish peroxidase (HRP)-conjugated HTLV-I/-II chimeric antigen and 3,3′,5,5′-tetramethylbenzidine (TMB) substrate solution.

### Enzyme linked immunosorbent assay (ELISA).

The ELISA plate wells were coated with RA (5–30 μg/ml) in sodium bicarbonate buffer (50 mM, pH 9.6) and incubated overnight at 4°C. The coated wells were washed twice with PBS and then blocked with blocking buffer (1% BSA in PBS with 1% Tween-20) for 2 h at room temperature. Then, 50 μl of the infected sera were added to the plates and incubated for 1 h at 37°C. The HRP-conjugated HTLV-I/-II-chimeric antigen was added to the plates and incubated for 1 h at 37°C. After washing for six times, TMB substrate solution was added and the absorbance was determined at 450 nm after 30 min. Bovine serum albumin (BSA) was used as a negative control under the same conditions.

## RESULTS

### RA design.

**Fig. 1.**
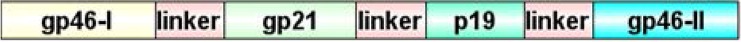
Graphical display of RA. The length of fragment (224 amino acids) and position of three linkers (EAAAK) are graphically illustrated.

EAAAK sequence was selected as the linker to retain the flexibility of the construct. Graphical display of RA has shown in Fig. 1. Codon usage bias in *E. coli* was improved by upgrading guanine-cytosine percent (GC%) and codon adaptation index (CAI) to 55.2% (GC% of *E. coli* is about 50) and 0.89 respectively. CAI of > 0.8 was regarded as good, in terms of high gene expression level (11). According to the sequence alignment results, we detected the sequence similarity between these protein amino acids in registered sequences.

### Construction of the expression vector.

The synthetic DNA encoding RA was sub-cloned into *BamHI* and *EcoRI* restriction sites of pGS21a (+) to generate pGS21a-RA. Accuracy of sub-cloning was confirmed by DNA sequencing.

### Expression of the recombinant RA.

**Fig. 2.**
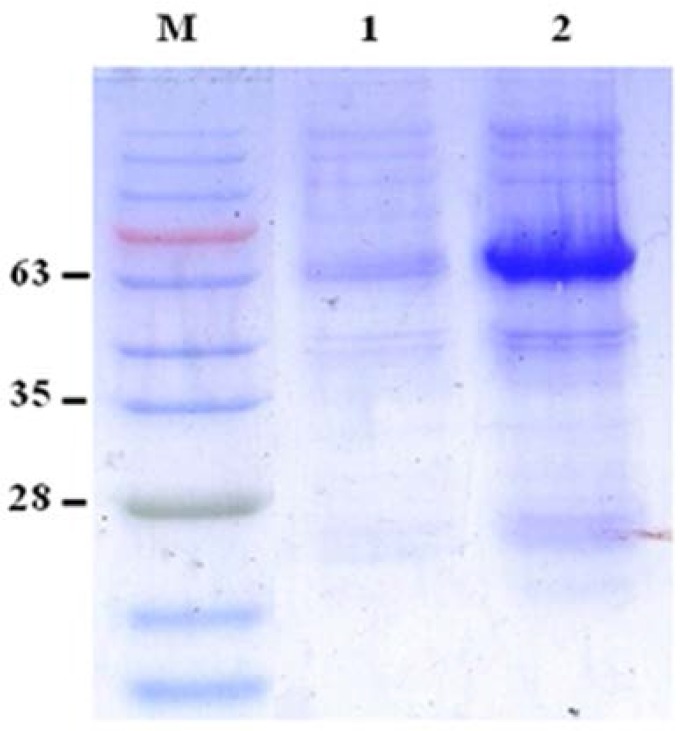
Expression of recombinant His-GST-RA in *E. coli* BL21 (DE3). Proteins were separated by SDS-PAGE (12% gel performed under reducing conditions). Lane M: protein molecular weight marker, lane 1: non-induced His-GST-RA/*E. coli* BL21 (DE3), Lane 2: induced His-GST-RA/*E. coli* BL21 (DE3).

The lysate of the induced recombinant *E. coli* BL21 (DE3) was analyzed by SDS-PAGE. The induction time was 12 h and the IPTG concentration was 0.4 mM. SDS-PAGE of the lysate of the induced pGS21a-RA/BL21 recombinant bacteria revealed a 63-kDa band, corresponding in size to the expected molecular weight of His-GST-RA (Fig. 2). However, no such band was observed for the induced pGS21a/*E. coli* transformants. This vector gave excellent inducible expression of RA, which accounted for approximately 30% of the total cellular proteins, as determined by thin-layer protein scanning of Coomassie blue stained SDS-PAGE gels.

### Solubility determination.

**Fig. 3.**
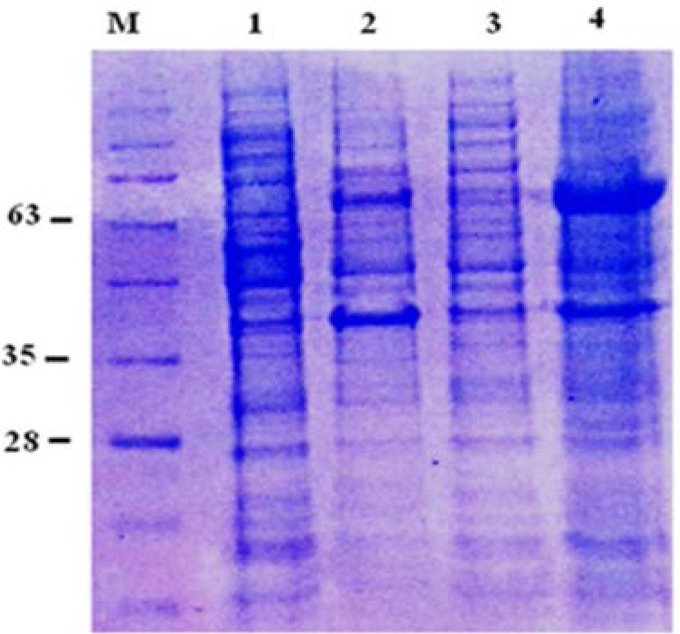
Solubility of the recombinant His-GST-RA. Samples were submitted to SDS-PAGE 12% and were stained with Coomassie blue G-250. Samples were directly resus-pended in SDS-loading buffer and boiled for 10min. Lane M: protein molecular weight marker; lane 1: non-induced *E. coli* BL21 (DE3), lane 2: *E. coli* BL21 (DE3) induced with 0.4mM IPTG; lane 3: pellet of *E. coli* BL21 (DE3) induced with 0.4mM IPTG lane 4: supernatant of *E. coli* BL21 (DE3) induced with 0.4mM IPTG.

To check the distribution of the protein expressed in the soluble and insoluble fractions under optimized conditions, the supernatant and pellet of the cell lysate were analyzed by SDS-PAGE. Analysis of the supernatant revealed a band with the expected molecular weight (63 kDa), whereas no visible band was noted upon examining the pellet of the cell lysate (Fig. 3).

### Simple purification of His-GST-RA.

**Fig. 4.**
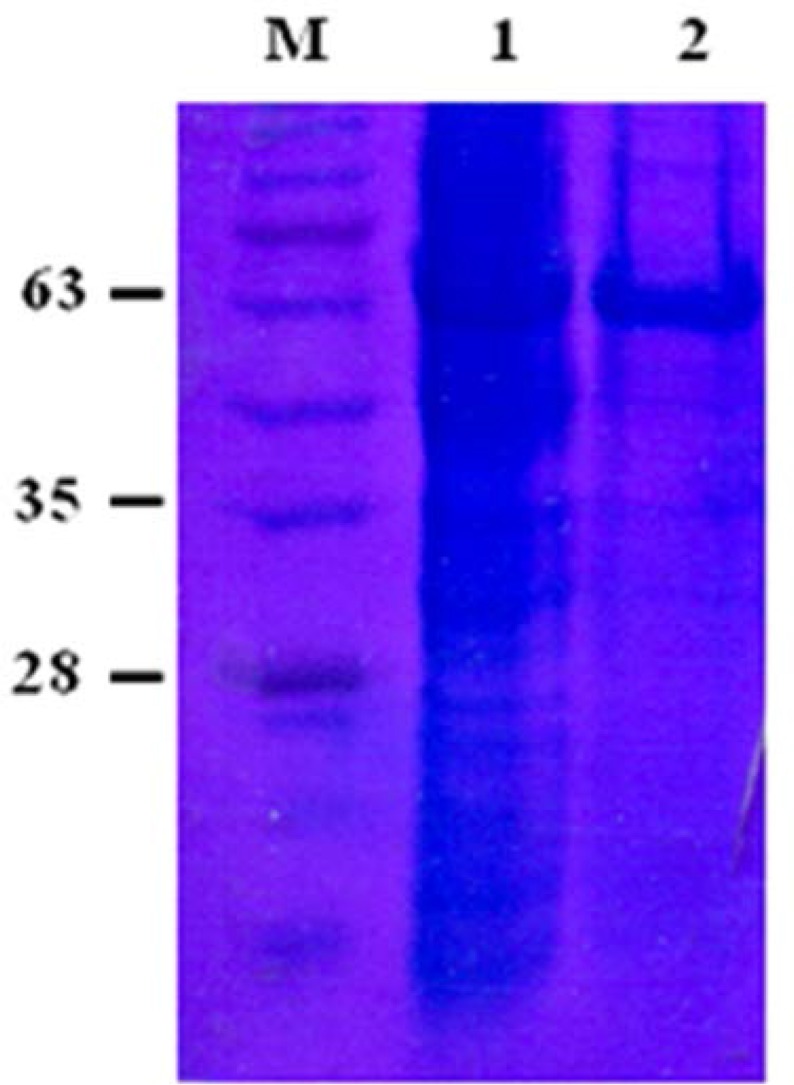
Purification of the expressed His-GST-RA. Proteins were separated by SDS-PAGE and stained by Commasie blue method. Lane M: protein molecular weight marker, lane 1: induced *E.coli* supernatant, lane 2: purified protein by Ni-chelating chromatography.

The His-GST-tagged RA was purified under native conditions using Ni-NTA agarose beads. The protein was visualized as a 63-kD band and its purity was around 90% (Fig. 4).

### Characterization of the recombinant His-GSTRA.

**Fig. 5.**
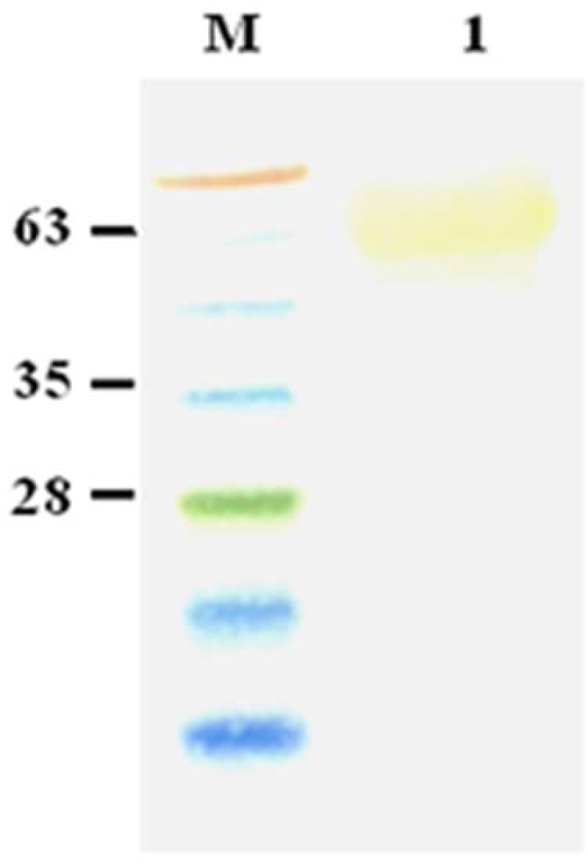
Western blotting of purified His-GST-RA with HTLV-I/-II infected sera. Lane M: molecular weight marker and lane 1: purified His-GST-RA.

Using HTLV-I/-II infected sera, the binding characteristic of the His-GST-RA was confirmed by western blot analysis. As expected, the 63-kD band exhibited affinity to the antibodies (Fig. 5).

### Binding activity of the purified His-GST-RA to HTLV infected serum antibodies.

**Fig. 6.**
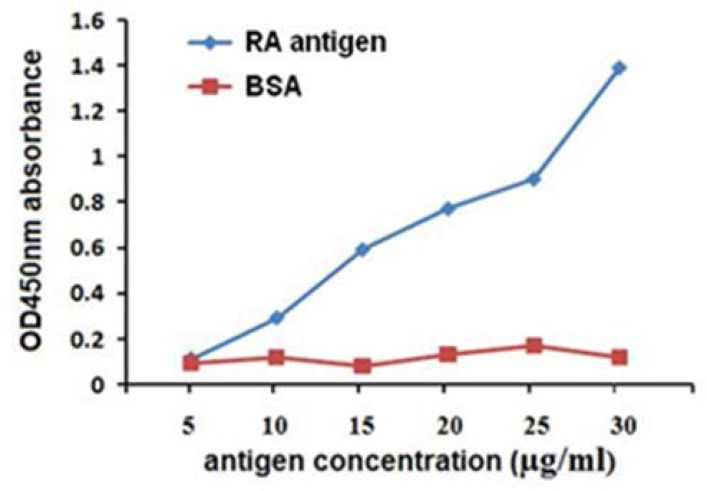
Binding activity of active His-GST-RA to infected sera. ELISA assay demonstrates a binding curve at various concentrations of active His-GST-RA to serum antibodies. BSA was used as negative control under the same condition.

The binding activity of the His-GST-RA to the infected serum antibodies was determined by ELISA. As shown in Fig. 6, the active protein bound to anti-HTLV-I/-II antibodies present in the pooled infected serum in a concentration-dependent manner, the signals increased as more RA coated. Also, comparison of RA ELISA results with commercial ELISA results indicated that these interactions were highly specific (Table 1). ELISA results indicated that RA peptide also show high absorbance at OD450nm in comparison to commercial kit. This may be related to high immunodominant epitopes present on RA antigen. Healthy controls did not show significant ELISA reactivity.

**Table 1.** Results of HTLV-I/-II antibodies detection in 10 infected serum samples.

**Number**	**Commercial kit**	**His-GST-RA ELISA**
1	1.56	1.65
2	2.10	1.31
3	1.03	1.24
4	1.82	1.93
5	1.35	1.75
6	1.97	1.59
7	1.73	1.34
8	1.67	1.66
9	1.23	1.46
10	1.70	1.86

## DISCUSSION

HTLV-I and HTLV-II are major causes of neurologic disease and lymphocytic leukemia. Peptides derived from gp46-I, gp46-II, gp21 and p19 are important antigens that are widely used in the serological tests of HTLV infection (12). The present study aimed to develop a simple method for the expression and purification of chimeric antigen that contains these peptides.

To obtain high quantity of RA, pGS21a was chosen as the expression vector and *E. coli* was selected as the host, owing to advantages such as low cost, ease of use, high level of productivity, and presence of an N-terminal His-GST-tag in the vector pGS21a, which allows easy purification (13, 14).

Many factors, including codon bias of *E. coli*, the GC content of the foreign gene and growth conditions, influence foreign gene expression (15, 16). Codon contents of the viral protein sequence are rarely used in prokaryotic cells. Mass production of proteins from sequences containing rare codons is difficult, because the tRNAs that encode these codons are rapidly depleted during over expression (14). To maximize the level of expression, we optimized the gene of interest by substituting the rare codons with more preferred ones.

An ideally optimized gene would show CAI of 1, however even though no *E. coli* gene reaches to this theoretical value (11). The optimized RA gene had a CAI of 0.87, indicating that the optimized gene could be over expressed (11). Furthermore G/C ratio of optimized gene was 55.2% and this reported to be associated with over-expression. As expected from gene optimization results, His-GST-RA was efficiently expressed in *E. coli*. We believe that elimination of rare codons by optimizing the gene of interest favored this increased expression, because these factors potentially hindered translation.

Over expression of foreign proteins in *E. coli* often leads to the formation of inclusion bodies (17). Expression of proteins as inclusion bodies has several advantages, such as simple isolation, resistance to proteolysis, high-level expression, and simplicity of monitoring by SDS-PAGE, thus making it an attractive approach for recombinant protein production (18). However refolding of inclusion body is complicate and time consuming and need to optimize for any protein (14). It has been suggested that low induction temperature, low IPTG concentration and solubility tags could improve the solubility of the proteins (13). In this study, we use 0.4mM IPTG, and 18°C for expression of RA. As indicated by SDS-PAGE, under this condition *E. coli* expressed the RA in soluble form.

The soluble proteins were purified using affinity chromatography under native condition. SDS-PAGE revealed that the purity of the His-GST-RA was approximately 90% after purification. The final yield of the active His-GST-RA was 25 mg per liter of bacterial culture. Further identification of antigenic properties of the recombinant protein was confirmed by western blotting.

A few studies have shown antibodies against GST tag present in serum of healthy individuals and may give false positive results in ELISA (19). Thus we used a HTLV-I/-II chimeric protein conjugated to HRP instead of anti-human IgG for detection of interacted antibodies.

The results of ELISA confirmed that His-GST-RA derived from *E. coli* displayed good immunoreactivity with anti-HTLV-I/-II antibodies in the infected human sera. Zarnagh et al. indicated that chimeric antigen including various epitopes is effective for detection of HBV and HTLV-1 antibodies and can be used for screening (12).

The results obtained in this study are in agreement with those reported by Viscidi et al. (20). They used synthetic peptides from HTLV-I (ENV-I), HTLV-II (ENV-II) and transmembrane protein (TM) for sero-diagnosis of HTLV and discrimination of HTLV-I from HTLV- II infections. Their results indicated that the synthetic peptides may be a useful candidate for diagnosis of infections (20). In another study Mosadeghi et al. used the purified recombinant HTLV-I protein (His-GST-p19) and reacted with HTLV-I antibodies. They reported that specificity of strong reactivity of p19 was compatible to the ELISA results of p19 fusion peptide (14). These studies support our results. To evaluate the specificity of the method, serum samples from patients, plus healthy controls, were involved in this study. Detection results of 20 clinical serum samples were confirmed positive for HTLV and 20 samples were HTLV negative, by RA-ELISA indicating high sensitivity and specificity of the test. Sera from healthy controls did not display any recognition of RA. No cross-reactivity between positive sera and healthy controls was detected. These data demonstrated that this antigen was specifically reactive towards anti-HTLV-I/-II. The sequence alignment results also estimated that the designed linear peptide had nucleotide similarity with other epitopes isolated HTLV-I/-II strains from different regions of the worlds.

Due to the low number of infected and healthy sera, we recommended additional screening with more specimens to confirm sero-diagnosis effect of RA antigen.

In summary, our findings demonstrated that RA probably is suitable for anti-HTLV-I/-II screening assay and could possibly be an alternative to *E. coli*-derived fusion peptides to obviate separate antigen preparation, but needs further investigations.

## CONCLUSION

In this study, a recombinant antigen (RA) was constructed to be used in ELISA for screening the suspected cases for HTLV infection. Integration of the GST-fusion, low temperature induction and low IPTG concentration are excellent strategies for expressing this antigen as soluble form in *E. coli* cells. The GST fused protein retained the unique antigen–antibody interaction property. The system optimized in this study could allow feasible and cost-effective preparation of the RA antigen.
